# Protein Adducts and Protein Oxidation as Molecular Mechanisms of Flavonoid Bioactivity

**DOI:** 10.3390/molecules26165102

**Published:** 2021-08-23

**Authors:** P. Matthew Joyner

**Affiliations:** Natural Science Division, Pepperdine University, 24255 Pacific Coast Highway, Malibu, CA 90263, USA; matt.joyner@pepperdine.edu

**Keywords:** flavonoids, protein adducts, quinones, quercetin, EGCG, flavonoid bioactivity, flavonoid biological mechanisms, protein-flavonoid interactions

## Abstract

There are tens of thousands of scientific papers about flavonoids and their impacts on human health. However, despite the vast amount of energy that has been put toward studying these compounds, a unified molecular mechanism that explains their bioactivity remains elusive. One contributing factor to the absence of a general mechanistic explanation of their bioactivity is the complexity of flavonoid chemistry in aqueous solutions at neutral pH. Flavonoids have acidic protons, are redox active, and frequently auto-oxidize to produce an array of degradation products including electrophilic quinones. Flavonoids are also known to interact with specificity and high affinity with a variety of proteins, and there is evidence that some of these interactions may be covalent. This review summarizes the mechanisms of flavonoid oxidation in aqueous solutions at neutral pH and proposes the formation of protein-flavonoid adducts or flavonoid-induced protein oxidation as putative mechanisms of flavonoid bioactivity in cells. Nucleophilic residues in proteins may be able to form covalent bonds with flavonoid quinones; alternatively, specific amino acid residues such as cysteine, methionine, or tyrosine in proteins could be oxidized by flavonoids. In either case, these protein-flavonoid interactions would likely occur at specific binding sites and the formation of these types of products could effectively explain how flavonoids modify proteins in cells to induce downstream biochemical and cellular changes.

## 1. Introduction

Flavonoids are a ubiquitous and numerous group of plant secondary metabolites with hydroxylated phenyl rings that include the flavones, catechins, and anthocyanins. A wide variety of hydroxylation, methyoxylation, glycosylation, and oligomerization patterns have been described for this class of compounds, and thousands of unique polyphenol structures have been reported in the literature [1,2]. Polyphenols continue to intrigue the scientific community due to the extensive variety of biological activities associated with these compounds, including benefits to cardiovascular health [3], neuroprotection [4], and cancer prevention [5,6]. Many polyphenols have been reported to possess antibacterial activity [7], and the specific biological targets of some of these compounds have been found to include inhibition of nucleic acid synthesis, energy metabolism, cell wall synthesis, and fatty acid biosynthesis [7,8].

A SciFinder Scholar search on 25 June 2021 on the term “flavonoid” with the search limited to journal records yielded ~79,000 results. The term “catechin” yielded ~36,000 results and the term “polyphenol” yielded ~50,200 results. The vast number of peer-reviewed publications related to these terms demonstrates the broad interest of the scientific community in this class of compounds as well as the challenges of attempting to summarize information related to this topic. Due to the very large number of papers related to this topic, I will not attempt to create an exhaustive review. As far back as the year 2000, there was already sufficient material for an excellent review to be published by Middleton et al., with more than 1000 references [9]. In consideration of the large number of papers related to flavonoids published since then, I will instead attempt to summarize the results from highly cited papers that provide new insights into the molecular mechanisms of flavonoids in humans.

Despite the extensive body of primary scientific literature covering flavonoids, progress has been slow towards understanding the molecular mechanisms of how these compounds may exert biological effects in humans. The radical-scavenging activity of flavonoids, commonly called their “antioxidant” activity, has been widely hypothesized as a mode of action to explain the many observed biological effects of these compounds. A highly cited review article from the year 2000 states that “[p]olyphenols exhibit a wide range of biological effects as a consequence of their antioxidant properties” [10]. More than 20 years later, a recent meta-analysis states that “[f]lavonoids have been hypothesized to exert beneficial effects towards the cardiovascular system through their antioxidant and antiradical action” [11]. This “antioxidant theory” in the scientific literature has persisted, despite extensive evidence that the low bioavailability of dietary polyphenols as well as a general absence of direct evidence of radical scavenging activity in cells makes it very unlikely that the beneficial effects of these ubiquitous compounds is due to their so-called antioxidant properties [12,13,14]. Unfortunately, papers are still being published that report the isolation of phenolic metabolites from various plant sources and use a rationale of antioxidant activity based on radical-scavenging assays as a justification for some proposed health benefit. However, the concentration of flavonoids and their colonic metabolites in human plasma is around five-fold less than the concentration of endogenous radical-scavenging metabolites such as α-tocopherol or ascorbic acid [15,16,17]. The majority of consumed dietary flavonoids are absorbed from the small intestine as degradative metabolites after being metabolized by gut microbes [16,18], which strongly supports the conclusion that dietary flavonoids simply will not accumulate in most human tissue at sufficient concentrations for their radical scavenging activity to be relevant.

In short, the antioxidant theory of flavonoid bioactivity needs to be retired. Responsible editors and referees should recommend rejecting new papers that attempt to invoke the antioxidant theory as justification of performing radical-scavenging assays unless specific, compelling evidence of this activity in vivo is presented to support such a justification. Additional care must be taken by editors and referees to identify experiments that fail to account for known artefacts that can arise from flavonoid studies, such as their propensity to generate reactive oxygen species in cell culture media [19,20]. The scientific community must not continue to be complicit in the publication of peer-reviewed studies that persist in advancing a theory that is clearly contradicted by the majority of well-designed studies on the subject or that propagate unsound experimental methods.

Thus, if the antioxidant theory is baseless, how can the observed bioactivity of flavonoids be explained? Fortunately, there is an extensive body of literature to draw from to generate alternative hypotheses. A rapidly growing body of evidence suggests that dietary polyphenols may exert their beneficial effects in humans by modulating the gut microbiome [21,22,23]. Although there are many reports of various flavonoid treatments causing significant changes in the gut microbiome of both humans and rodents [22,24,25,26], there is still no molecular-level causal explanation for these observations. Possible mechanisms of flavonoid bioactivity could include growth inhibition of gut microbes [7,21,27] or inhibition of bacterial lipid metabolism [28,29]. Establishing a molecular-level mechanism for flavonoid biological activity in bacteria will be a dramatic step forward in determining the effects of these compounds on human health. 

**Figure 1:**
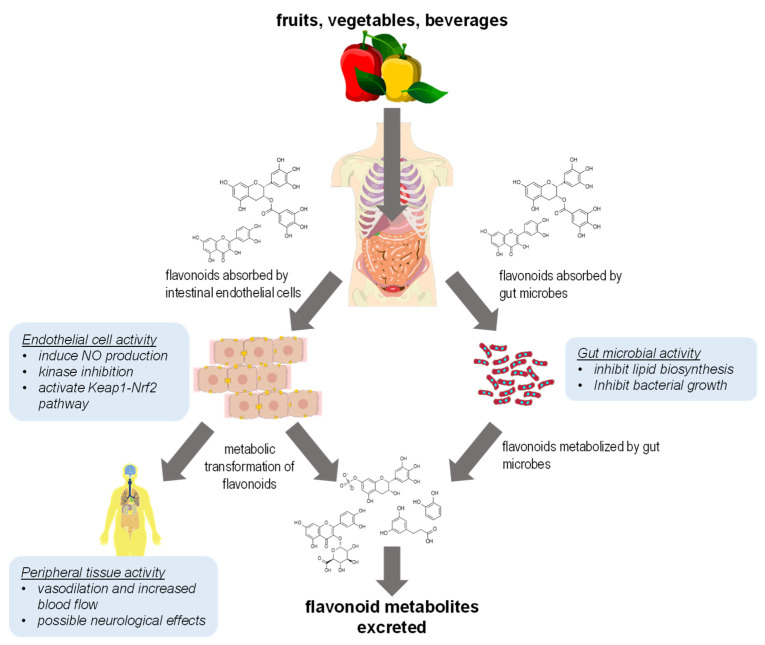
A summary of the most reasonable primary routes of flavonoid bioactivity in humans. Flavonoids will exhibit most of their bioactivity in intestinal epithelial cells and in gut microbial cells. After metabolic degradation by intestinal microflora, microbial metabolites of flavonoids are absorbed by the intestines and excreted.

There are reports that consumption of dietary flavonoids can increase blood flow in the peripheral vascular system and that this phenomenon can cause a corresponding increase in cerebral blood flow (reviewed in [30,31]). Flavonoids may be able to induce production of nitric oxide in endothelial tissue, causing vasodilation and thus indirectly improving cerebral blood flow [30,32,33]. These results demonstrate that some health benefits of dietary flavonoid consumption may arise from secondary physiological effects rather than direct interaction by flavonoids with cellular targets, which adds to the complexity of investigating flavonoid bioactivity. Although bioavailability of dietary flavonoids is very poor, there still exists some conflicting evidence that flavonoids may be able to accumulate in human tissues [30], thus holding open the possibility of direct effects on various human physiological systems. However, the bulk of the evidence supports the hypothesis that dietary flavonoids exert effects in humans primarily through their activity in the intestinal system by either modifying commensal gut microbes or in intestinal epithelial tissue lining the gut (Figure 1). Therefore, a new hypothesis is needed to explain the bioactivity of flavonoids at the biochemical/molecular level within this context.

In general, flavonoids must induce cellular changes by interacting with one of the four general categories of biomolecules—nucleic acids, proteins, lipids or carbohydrates. While there is clear evidence that flavonoids can form covalent adducts with DNA [34,35] and induce oxidative damage in DNA [36], I will not consider this as a plausible explanation for most of the observed flavonoid bioactivities since it would require flavonoids to migrate through cells to a nucleus in sufficient quantity to damage DNA. Flavonoid binding to transcription factors could potentially increase relative flavonoid concentration in the nucleus, thus making interacting with DNA more probable. While not impossible, this is still not the most plausible explanation due to the lower probability of flavonoid-DNA interactions occurring and the general absence in the literature for evidence of flavonoid-DNA interactions in vivo. The non-polar properties of flavonoid aglycones make the interaction between lipids and flavonoids highly probable in cells, and there are intriguing results that support flavonoid–lipid interactions as a mechanism for inducing changes in cellular signaling and metabolism [37,38,39]. However, it becomes extremely difficult to propose a mechanism that would connect flavonoid–lipid interactions with many of the observed biological effects on proteins and cytosolic cellular components, and it will not be considered further in this review. I am not aware of any reports of direct flavonoid-carbohydrate interactions connected with bioactivity, and it is difficult at this point to imagine how this type of interaction could explain flavonoid bioactivity. The remaining and most probable specific target for flavonoid bioactivity is through specific interactions with proteins, and there are many studies that support the hypothesis that flavonoid–protein interactions are a crucial component of many of the known cellular effects of flavonoids [9,40,41,42].

There have been multiple reports that describe the affinity and binding interactions of some flavonoids with lipophilic carrier proteins such as β-lactoglobulin, albumin and casein [43,44,45,46,47,48,49,50,51], salivary proteins [52,53,54], or beverage proteins [55,56,57], and, in most cases, these interactions appear to be non-specific. Flavonoids are known to bind to and inhibit many kinases (reviewed in [9,41]), paraoxonase 1 [58,59] α-amylase [60,61,62], α-glucosidase [63], and furin [64], enoyl-ACP reductase [28], TrkB [65], fructose-1,6-bisphosphatase [66], and p68 [67]. The large number of reports of protein-flavonoid interactions makes it impractical to cite all relevant examples. Despite this extensive body of research, there is not a unified molecular mechanism that has yet been proposed that could explain all (or at least most) of these observed bioactivities.

Although the redox activity of flavonoids is widely believed to be relevant to their bioactivities, it remains uncertain how this redox activity could act with specificity on so many biological targets since radical scavenging would not be expected to target specific proteins in most cases. I propose that the formation of protein adducts with flavonoids and the oxidation of specific amino acid residues in proteins could provide a mechanistic explanation for many of the cellular changes induced by flavonoids. The oxidation of specific amino acid residues such as cysteine, methionine, tyrosine and proline has been demonstrated to play important roles in regulation and signaling in a variety of cellular processes [68,69,70], and various electrophilic quinones are known to form adducts with proteins [71,72]. In this review, I will summarize the evidence that supports the hypothesis that flavonoid bioactivity arises from protein-flavonoid adducts and oxidative changes to proteins induced by flavonoids. I will describe the structures of reactive quinones of quercetin and (−)-epigallocatechin gallate (EGCG) and use these representative flavonoids to describe the chemical mechanisms of protein-flavonoid adduct formation or flavonoid-induced oxidative changes in proteins as well as the analytical methods needed to detect these protein products. This review is not intended to provide an exhaustive summary of all papers relevant to protein-flavonoid interactions. Instead, I will use examples from selected papers that provide useful examples of the recurring themes that occur throughout the protein-flavonoid literature.

## 2. Flavonoid Chemical Structures

Flavonoids are a broad category of plant metabolites with representative compounds found across the entire plant kingdom [73]. The number of unique chemical structures that can be classified as flavonoids is vast, and it is estimated that more than 10,000 unique flavonoid structures have been reported in the literature [2,74,75]. Many excellent reviews of the chemistry of plant polyphenols are available [2,7,8,76], so only a general summary of the most important features and representative structures will be provided in the following paragraphs.

**Figure 2:**
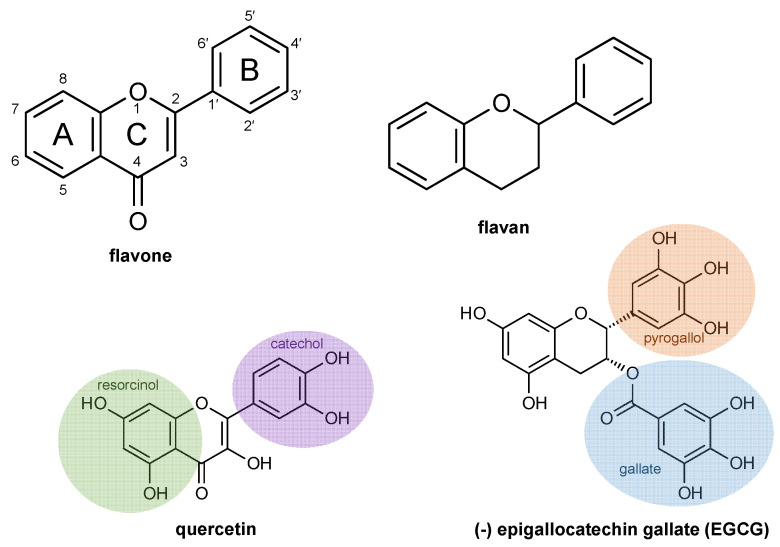
The core flavonoid scaffold for flavones and flavan along with the structures of the representative flavone quercetin and the flavan-3-ol EGCG.

The general chemical structure of flavonoids is composed of two phenyl rings connected by a heterocyclic ring (Figure 2). Flavonoids can be divided into a variety of categories, with flavones and flavans being two of the most commonly studied categories. Many flavonoids are glycosylated with one or more carbohydrates, which adds further complexity and diversity to their chemical structures. However, flavonoid glycosides are rapidly hydrolyzed in the human gut so the aglycone structures are expected to have the greatest relevance to human health [77]. Quercetin is one of the most frequently studied flavonoids because it is a major component of many different plant metabolomes, and it is active in most in vitro and in vivo bioassays. Quercetin is a flavonol with an unsaturated C2–C3 bond, giving it a planar structure and a conjugated pi-system that enables the delocalization of electrons across the B- and C-rings. The catechins are flavan-3-ols that are especially common components of plants used for teas or as herbal remedies by many human populations, with EGCG being one of the most frequently studied compounds. The catechins have a saturated bond at C2–C3 which creates greater structural complexity compared to the flavones such as quercetin since it introduces two chiral centers and a non-planar structure. Because they have been studied so frequently and in such a broad variety of biological contexts and because they capture most of the chemical features found in a huge number of flavonoids, quercetin and EGCG will be used as model flavonoids throughout this review.

Quercetin is a good representative for many flavonoids since it possesses the 3′,4′-dihydroxy B-ring (catechol), the 5,7-dihydroxy A-ring (resorcinol), unsaturated C2–C3 bond (flavone), and the oxychromen-4-one rings (A- and C-rings). EGCG is a good representative for many flavonoids because it possesses the 3′,4′,5′-trihydroxy B-ring (pyrogallol), the 5,7-dihydroxy A-ring (resorcinol), saturated C2–C3 bond (flavan), the chromene moeity (A- and C-rings), and the 3′,4′,5′-trihydroxy (gallate) substituent at C3. An incredible variety of flavonoids possess some combination of these chemical features, often with small differences in hydroxylation patterns, methoxy groups or glycosylation patterns [1,2,7,74].

It is apparent from this brief summary that there are a staggering number of unique chemical compounds that can be placed under the general term of flavonoids. This chemical diversity makes it difficult to imagine that a single mechanism of action could explain the equally diverse range of biological and pharmacological activities attributed to these compounds—and yet one property that has been consistently identified with the vast majority of these compounds is their redox activity.

## 3. Chemistry of Flavonoids in Aqueous Solutions

One of the challenges of flavonoid research is actually the low barrier of entry to performing experiments with flavonoids. Many flavonoid compounds are readily available from a variety of commercial sources and anyone can buy a series of flavonoids and add them to cells or enzymes and observe interesting effects. Despite this relative ease of experimentation, however, the chemistry of flavonoids in aqueous systems is very complex and difficult to control and can easily lead to the generation of results from cell culture or enzyme assays that may not have real biological significance. These challenging chemical properties include poor solubility, formation of complex protic equilibria, auto-oxidation, and redox cycling with other experimental components. As a result of this complexity, many studies of flavonoid bioactivity may provide almost no insights into what may actually happen in humans when we consume dietary flavonoids. If the scientific community is going to make meaningful progress in understanding the physiological effects of these ubiquitous compounds, we must all do a better job of accounting for flavonoid chemistry in our experiments. This section will provide an overview of the known chemistry of flavonoids in aqueous solutions at physiological pH.

Although many studies report the relative importance of the formation of anions by deprotonation of flavonoid hydroxyl groups, there is a surprisingly wide range of pK_a_ values reported for these ionizable hydroxyl groups [78,79,80]. Just for quercetin, the first pK_a_ value has been reported to be as low as 3.3 and as high as 8.4 in aqueous solutions [80], which is a 100,000-fold range! The most reliable studies agree on values for the first three pK_a_’s as 8.4, 9.3, and 11.0 in aqueous solutions [78,80]. The 7-OH of the A-ring is the first position to be deprotonated for flavones in aqueous solutions, followed by the 4′-OH and then the 3-OH [78]. This order of relative acidity is dependent on the overall hydroxylation pattern on the flavonoid, but, for many flavonoids, this pattern should hold true. The formation of the anionic flavonoid is important because it is more easily oxidized than the neutral species [78,81,82,83]. If the measured pK_a_ of 8.4 for the 7-OH group is accurate, then a mole ratio of approximately 99:1 for neutral:anion species of flavonoids with 7-OH substituents would be expected in aqueous solutions at physiological pH (pH 7.4). Although this ratio greatly favors the neutral species, enough anions may be present to create interactions with other biomolecules that are distinct from the neutral species and have meaningful biological consequences, similar to how cysteine residues (pK_a_ 8.3) can be deprotonated to reactive thiolates in various proteins.

It is widely understood that flavonoids are redox active and can spontaneously oxidize in aqueous solutions. However, the specifics of flavonoid redox chemistry can be quite complex and may be an often overlooked confounding variable in in vitro and in vivo bioactivity experiments. There are likely some differences in redox reactivity that depends on the unsaturation of the C2–C3 bond since the resulting pi-conjugation creates a mostly planar molecule with the ability to delocalize electrons across the B- and C-rings. However, despite the unique properties that depend on the C2–C3 bond structure, there appears to be a general mechanism of oxidation that can be applied to a wide variety of flavonoids. Supporting evidence for this mechanism is available from studies of both flavan-3-ols (catechins) and flavones (quercetin).

In a study of a series of catechins, the authors report that the B-ring hydroxyl groups first react with dioxygen, oxidizing the catechin to the semiquinone and reducing the oxygen to superoxide anion radical [83]. The superoxide anion radical then reacts with the semiquinone or another fully reduced catechin to yield hydrogen peroxide and either another seminquinone or the fully oxidized quinone product. This observation held for epicatechin, epigallocatechin, epicatechin gallate, and EGCG, suggesting it is a general mechanism for flavonoids with either dihydroxy or trihydroxy rings. The reactivity of catechins with hydrogen peroxide was observed to be essentially zero, indicating that they do not scavenge all reactive oxygen species (ROS) from aqueous solutions but react selectively with a superoxide anion radical. Hydronium was observed to quench the reaction between catechin and superoxide, indicating an important role for the flavonoid anion in this mechanism. Another study of the superoxide scavenging activity of catechins found that, while almost all flavonoids examined were able to scavenge superoxide radical anions, the flavan-3-ols with a dihydroxy substituted system had much lower scavenging activity than either flavan-3-ols with trihydroxy substituted rings (either gallate or pyrogallol) or flavones with dihydroxy substituted B-rings [84]. The conjugated system of flavones such as quercetin would be expected to follow the same mechanism as the catechins and be able to both generate and scavenge superoxide ions in aqueous solutions, just like the catechins [85].

Investigation of the radical scavenging mechanism of a series of flavones showed that their anionic forms are oxidized predominantly via the sequential proton-loss electron transfer (SPLET) mechanism while the neutral species is oxidized via a hydrogen atom transfer (HAT)/proton coupled electron transfer (PCET) mechanism [78]. The SPLET mechanism was found to be orders of magnitude faster than the HAT/PCET mechanism for many of the flavones studied, with the rate enhancement being dependent on the ability of the flavonoid to donate a proton to the solvent.

**Figure 3:**
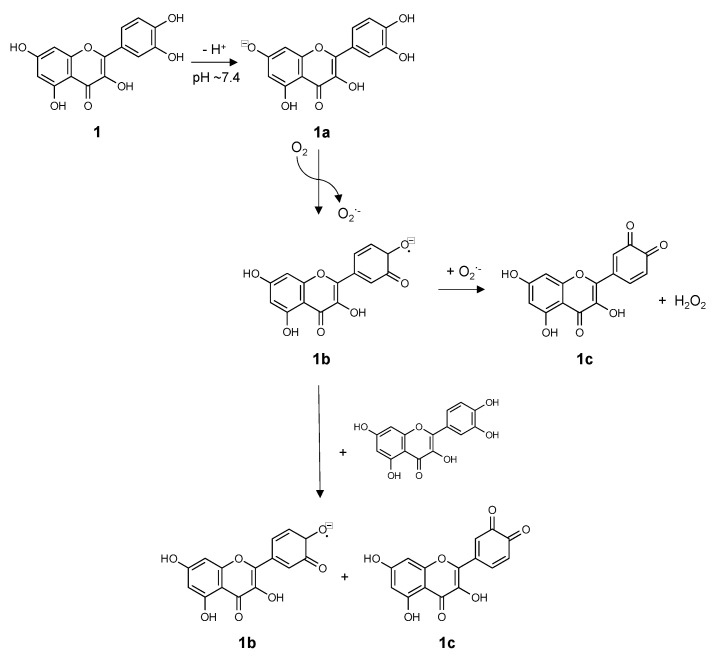
A proposed general auto-oxidative mechanism for flavonoids using quercetin as a representative structure.

Putting the findings of [78] and [83] together suggests the following general mechanism of flavonoid auto-oxidation in aqueous solutions (Figure 3). First, the flavonoid (**1**) is deprotonated by the solvent, followed by transfer of an electron from the anionic phenolate (**1a**) to molecular oxygen, yielding superoxide and a flavonoid semiquinone radical(**1b**). The semiquinone radical will initially be located at the site of the phenolate (i.e. C7 or C4′), but, for flavonoids with a catechol group on the B-ring, rapid charge transfer and rearrangement will move the radical to the catechol position. The semiquinone radical (**1b**) will then be oxidized by either the superoxide anion radical or another fully reduced flavonoid to yield hydrogen peroxide and either the fully oxidized quinone (**1c**) product or a semiquinone radical, respectively. There is evidence that the flavonoid radical will undergo disproportionation [83,86], providing multiple possible routes for propagation of the radical. The evaluation of HAT/PCET vs. SPLET mechanisms for electron transfer from the flavonoid were performed in organic solvents with DPPH· as the radical partner [78], so it is unclear which mechanism the second electron transfer would utilize in aqueous solutions. Regardless of the mechanistic details that remain to be determined, this reaction scheme is consistent with experiments using electrochemical methods that consistently find that most common flavonoids are oxidized to quinones via a two electron, two proton mechanism [81,82,87,88,89]. All of these studies provide further evidence illustrating the necessity of taking pH and flavonoid deprotonation into account when evaluating flavonoids in in vitro or in vivo bioassays.

**Figure 4:**
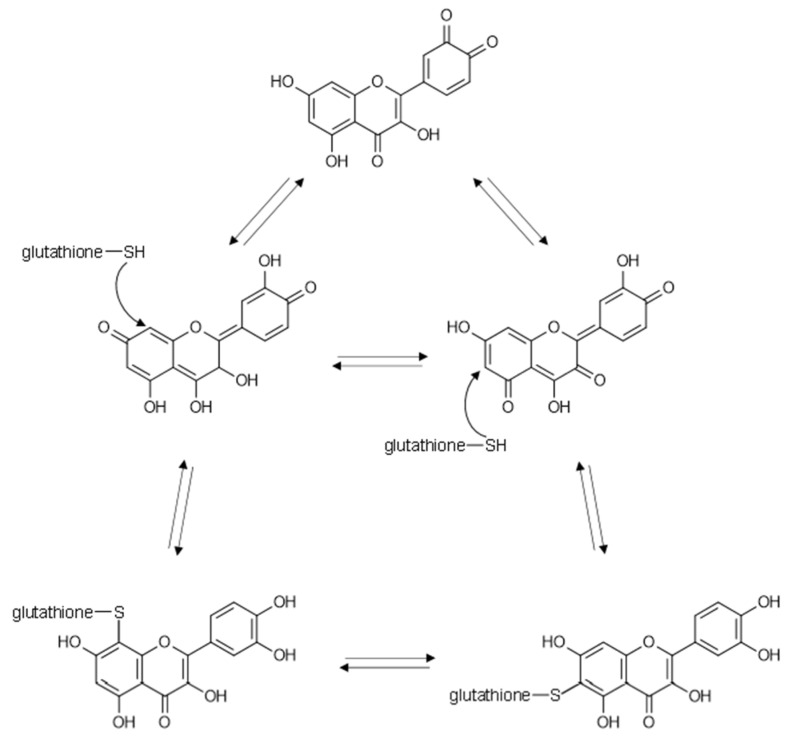
Glutathione forms flavonoid adducts via Michael addition to the quinone methide isomers of oxidized quercetin.

In the low oxygen environment of the human intestinal tract, the oxygen-dependent auto-oxidative pathway of flavonoids may be less relevant. An alternative pathway to generating oxidized products of flavonoids is via enzymatic oxidation by various peroxidases, tyrosinases or related enzymes in intestinal epithelial cells, commensal microbes or even enzymes from dietary fruits and vegetables. In a wonderfully cogent series of papers, Ivonne Rietjens and colleagues demonstrated that a variety of flavonoids are oxidized by tyrosinase and peroxidase enzymes, that the oxidized flavonoid products of these reactions possess in vivo bioactivity and that the bioactive oxidized products are produced in cells via enzymatic processes and can be inactivated through the formation of flavonoid-glutathione adducts [90,91,92,93,94,95]. Glutathione reacts with the highly electrophilic quinone methide isomers of quercetin and other flavonoids via a Michael addition; glutathione adds via its sulfhydryl group to the A-ring at C6 or C8 on quercetin and to the B-ring at C2′ or C6′ for EGCG and other pyrogallol-containing catechins [90,95] (Figure 4).

The flavonoid-peroxidase system has been reported to produce more than 20 distinct products [90]. This is consistent with electrochemical studies that demonstrate a kinetically controlled production of distinct oxidative products from quercetin [88]. After initial formation of the *o*-quinone on the B-ring of quercetin, a zero order mechanism leads to formation of a closed furan ring between the C- and B-rings. A first order mechanism involves nucleophilic attack by water at C6′ of the oxidized *o*-quinone, resulting in a *o*-hydroxy, *p*-quinone system on the B-ring; this B-ring system is predicted by the authors to be unstable and susceptible to formation of dimers, trimers and other degradation products [88].

When glutathione is added to the flavonoid-peroxidase system, only two major products were observed and both were glutathione adducts [90]. This indicates that essentially all of the observed oxidation products of quercetin arise from a central intermediate that is most likely the quinone methide based on density functional theory (DFT) calculations [91]. The 6-glutathionyl-quercetin and 8-glutathionyl-quercetin products are also in equilibrium with each other and equilibrate to a 55:45 ratio in aqueous solution [91], which indicates that glutathione-flavonoid conjugates are dynamic and therefore should not be assumed to be irreversible reaction products. There are multiple reports that demonstrate the attenuation of flavonoid inhibitory activities of various enzymes by the addition of glutathione [64,93,94,95,96]. The reproducibility of this phenomenon indicates that the bioactivity of flavonoids in many cases may arise from reactive quinone products of the flavonoids rather than the reduced phenolic structures typically considered for flavonoids.

Flavonoids are also known to chelate various transition metal cations commonly encountered in cells such as iron, copper, and others and can generate ROS such as H_2_O_2_ and flavonoid quinone products [97,98]. Halliwell and colleagues demonstrated that EGCG and other flavonoids can rapidly generate cytotoxic concentrations of H_2_O_2_ in cell culture media, and it is likely that this phenomenon depends on flavonoid-metal ion complexes [20,99]. This propensity of flavonoids to be oxidized by metal ions can be another confounding variable when investigating flavonoid biological mechanisms and should be accounted for through the use of careful controls or various ROS scavenging reagents or other methods.

It is troubling that so few papers related to flavonoid bioactivity appear to take these chemical properties of flavonoids under physiological conditions into account. This may be a major contributing factor to the relatively poor predictive utility of many existing flavonoid SAR models. If meaningful progress is to be made in identifying the chemical mechanisms of flavonoid bioactivities, then the scientific community must do a better job of accounting for their known chemistry and reactivity at physiological pH.

## 4. Flavonoids May Exert Bioactivity by Forming Reversible Protein Adducts

Any plausible hypothesis for flavonoid bioactivity needs to account for the observed specificity of flavonoid binding to various proteins. It has been known for more than 20 years that flavonoids are potent inhibitors of many kinases and other enzymes [9,41]. Investigations using ^14^C radiolabeled quercetin demonstrated extensive, irreversible protein-quercetin binding in cultured cells, and quercetin exhibited preferential binding to specific proteins [100]. Although the authors interpreted their results as evidence of covalent binding of the quercetin to specific proteins, no chemical or analytical evidence was presented to support that conclusion and none of the protein targets of quercetin were identified. Regardless, the report provides compelling evidence that quercetin (and probably other flavonoids as well) does bind with high affinity to specific proteins in cells. Human serum albumin has been proposed as a plausible protein target for flavonoids since it functions to bind and transport non-polar molecules through the circulatory system. Several studies have demonstrated that quercetin binds with specificity to both human and bovine serum albumin [51,101]. Another study showed that one or more products of oxidative degradation of quercetin are probably the molecules that bind to albumin since maximum binding to albumin was observed after oxidizing quercetin with peroxidase-H_2_O_2_, but this binding was completely eliminated when GSH was added to the mixture [50]. This result suggests that oxidation products of quercetin bind to albumin but not GSH-quercetin conjugates. Cys34 of human serum albumin is known to participate in ligand-binding interactions and to be a site of post-translational modification [102], so the probability of this residue forming a flavonoid adduct seems high. Considering the low bioavailability of flavonoids in plasma, flavonoid binding to albumin may not be biologically relevant, but it does provide further evidence that flavonoid–protein interactions often have specificity that is directly dependent on the redox activity of the flavonoids. 

That activation by flavonoids of the electrophile response element (EpRE)-mediated gene expression pathway in cell culture was shown to be attenuated by glutathioine-trapping of flavonoid quinones [93,94,95]. The EpRE pathway is activated by translocation of the Nrf2 transcription factor to the nucleus; under normal conditions, Nrf2 is continually ubiquinated and degraded due to interactions with the cytoplasmic protein Keap1 which recruit a ubiquitin E3 ligase and promote Nrf2 polyubiquitination [103,104]. Modification of key cysteine residues in Keap1 by ROS or electrophiles inhibit its ability to recruit the E3 ubiqutin ligase, which then allows Nrf2 to increase in concentration and promote expression of EpRE-related genes [103,104]. The EpRE inducement activity of flavonoids increased when glutathione (GSH) levels decreased and decreased when GSH levels increased, strongly supporting the hypothesis that flavonoid quinones activate EpRE-mediated gene expression by modifying protein targets [93,95]. Since Keap1 is known to be regulated by modification of cysteine residues, formation of Keap1 Cys-flavonoid adducts is a plausible mechanism for the activation of EpRE-mediated gene expression despite the current lack of direct experimental evidence.

Other important examples of protein-flavonoid interactions are the activity of 7,8-dihydroxyflavone acting as a specific and high affinity agonist of the BDNF receptor TrkB [65], inhibition of amylase and glucosidase by flavonoids [60,61,62,63,105], inhibition of paraoxonase 1 [58,59], and fructose-1,6-bisphosphatase [66] by flavonoids. This list is not intended to be exhaustive and certainly leaves out other relevant studies. The non-specific binding of flavonoids to salivary proteins has been well documented [52,53,54] and illustrates by contradiction the unique properties of flavonoid–protein interactions that are specific.

A common theme in all these studies is the difficulty of establishing meaningful structure–activity relationships based on non-covalent interactions. Although many of these reports do claim to identify SAR patterns, review of the identified structural features inevitably leaves the reader perplexed as it appears that, in most cases, the presence or absence of a single hydroxyl group dramatically alters the described activity to an extent that is simply not plausible based on typical non-covalent interactions observed between proteins and ligands. In at least one case, the development of a novel inhibitor intended to mimic the flavonoid scaffold yielded an inhibitory ligand that actually possessed a different binding mode than the flavonoids, despite the attempt to mimic its structural features [106]. The pattern of strange or inconsistent SARs throughout these reports strongly suggests that an alternative explanation to non-covalent protein-flavonoid interactions is needed.

As summarized previously in this review, many flavonoids are readily converted to reactive quinone methides in cells [40,90,91,92]. Therefore, it is important to consider the potential of these quinone methides and other reactive flavonoid redox products for their potential to act on cellular targets. Many examples exist of the formation of covalent protein adducts with quinones (reviewed by Chen and Li [71]), including the proteins cytochrome *c* [72], ribonuclease A [107], and neuroglobin [108]. There are even specific examples of the identification of covalent protein adducts with pyrogallol-containing catechins [109,110], although the detailed chemical features of those adducts were not elucidated. Altogether, this evidence strongly supports the hypothesis that flavonoids can modify protein structure and activity through the formation of covalent adducts. Modification of activity could occur by disrupting protein–protein interactions, as was predicted for cytochrome *c* [72], or by modifying active site residues as in the case of glyceraldehyde-3-phosphate dehydrogenase [109]. Nucleophilic amino acids such as cysteine and lysine are the most obvious candidates as sites for the formation of flavonoid adducts since the quinone auto-oxidation products of flavonoids will be strongly electrophilic. However, Fisher et al., described evidence for the formation of glutamate adducts as well through the formation of an ene-diol intermediate on the glutamate residue [72], which indicates that even weaker nucleophiles such as glutamate or aspartate may be able to form flavonoid adducts. Other amino acids with nucleophilic groups like tyrosine or histidine could also potentially be sites for flavonoid adduct formation. The idea that natural product derived electrophilic quinones could induce cellular bioactivity is not completely novel [71,111,112,113], but it has not yet been explicitly applied to flavonoids or gained widespread acceptance in the scientific literature.

**Figure 5:**
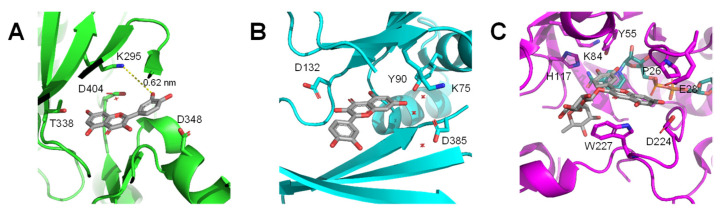
Interactions between flavonoid ligands and proteins from X-ray crystal structures; residues that could either form adducts or be oxidized by flavonoids are labeled. (**A**) the tyrosine kinase Hck (green) with quercetin (gray) (PDB 2HCK). The distance from K295 to the catechol group of quercetin is within a reasonable distance for electron transfer; (**B**) human inositol polyphosphate multikinase (IMPK; cyan) with quercetin (gray) (PDB 6M89); (**C**) prostaglandin F synthase (magenta) with NADPH (cyan) and the quercetin-3-*O*-glycoside rutin (gray). In all three images, water molecules are shown as red asterisks.

Reviewing X-ray crystal structures of protein–flavonoid complexes also supports the adduct hypothesis (Figure 5). A variety of flavonoids bind in the ATP-binding site of kinases and compete with ATP [106,114,115]. It is noteworthy that these crystal structures failed to reveal any clear structure–activity relationships that are consistent across a wide variety of flavonoid structures and some studies reported multiple, closely-related binding poses for the flavonoid ligands. In the structures of inositol polyphosphate kinases, there are lysine, glutamate, and tyrosine residues that either appear to directly interact with flavonoid ligands or are in very close proximity to the binding site [106,115]. The crystal structure of the tyrosine kinase Hck with quercetin also has lysine in close proximity to the ligand [114]. In the X-ray crystal structure of the enzyme prostaglandin F synthase with rutin (a glycosylated derivative of quercetin), there are tyrosine, histidine, and lysine residues directly interacting with or in very close proximity to the catechol ring of the flavonoid [116]. Considering the reactivity of the 6- and 8- positions of the quinone methide isomers of quercetin, all of these interactions would make the formation of covalent adducts plausible. It is also important to note that while these structures demonstrate apparent binding sites for quercetin it could be possible in some cases that formation of adducts at other residue outside of these observed binding locations could also induce the inhibitory activity observed in in vitro assays.

**Figure 6:**
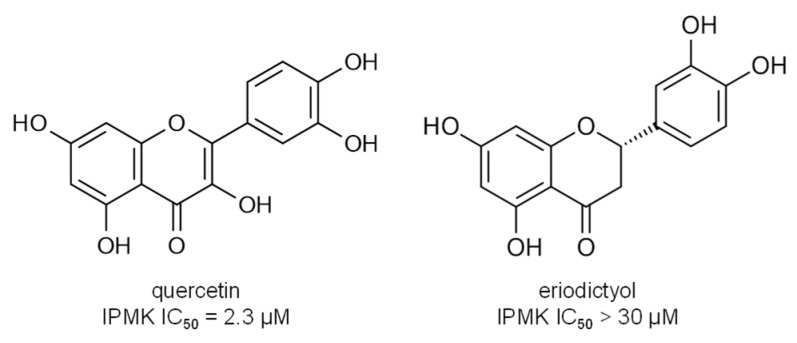
Comparison of structures of quercetin and eriodictyol and their inhibitory activity against human inositol polyphosphate multikinase (IPMK). IC_50_ values from [108].

Further evidence supporting the hypothesis that the inhibitory activity of flavonoids in these enzymes arises from reactivity with flavonoid quinones is that the proposed structure–activity relationship for flavonoids in the inositol kinase provides weak explanatory power for the observed changes in inhibitory activity. For example, there is a more than 10-fold difference in the reported inhibitory activity of quercetin and eriodictyol, despite the only structural differences being the absence of the 3-OH group in eriodictyol and the saturation of the C2–C3 bond [115] (Figure 5). These structural differences between quercetin and eriodictyol seem unlikely to cause the observed change in IC_50_ since the B-ring of quercetin has no observable direct interactions with side chains in the crystal structure. This pattern of a large change in inhibitory activity from only a small change in structure is common for the flavonoids and indicates that, in many cases, the inhibitory activity cannot be explained solely by non-covalent interactions with the protein (Figure 6). The loss of planarity of the molecule when the C2–C3 bond is saturated is often invoked as an explanation for some of these dramatic changes in inhibitor activity [62,63], but the loss of planarity seems likely to have more of an impact on the ability of the molecule to form reactive quinones due to differences in pi-conjugation patterns than it will on non-covalent interactions with proteins.

An important consideration in regards to this protein-flavonoid adduct hypothesis is the potential reversibility of these interactions. In studies where the authors concluded that flavonoids formed covalent bonds with proteins, the authors also described these interactions as irreversible [50,67,100,109,110]. However, in each case, the methods described do not provide sufficient evidence for true irreversibility; for example, none of these reports include attempts to wash or dialyze off the flavonoids from the protein targets in their methods. The glutathionyl–quercetin adducts described by Boersma et al., were shown to be in equilibrium with each other, indicating that formation of the thioether bond is reversible in aqueous solution at neutral pH [91]. This suggests that protein–flavonoid adducts may be reversible under cellular conditions despite being covalent, which would add an additional layer of complexity to designing experiments to evaluate these potential interactions.

Many studies have demonstrated that flavonoids are extensively metabolized after absorption in the intestine (reviewed in [77]). It is also firmly established that gut microbes degrade flavonoids into a variety of metabolic products that are absorbed into the blood stream and eventually excreted [15,16]. These products include sulfated and glucuronidated flavonoids from enzymes in human enterocytes as well as a variety of microbial products with hydroxylated phenyl groups. These metabolic products will have different physical and chemical properties from their flavonoid parents, but it is possible that, in some cases, they could still be oxidized to electrophilic quinones. For example, the major microbial flavonoid degradation product 5-(3′,4′-dihydroxyphenyl)-γ-valerolactone (3,4-diHPV) [15] still possesses a catechol group and therefore seems likely to be readily oxidized to a quinone. Because of the metabolic transformations that occur after passage of absorbed flavonoid from intestinal epithelium into the circulatory system, it seems probable that most of the flavonoid bioactivity will occur in these intestinal cells rather than in other tissues since the bulk of absorbed flavonoid metabolites are eventually excreted [16]. However, further studies are needed to test the adduct hypothesis on these flavonoid metabolites.

**Figure 7:**
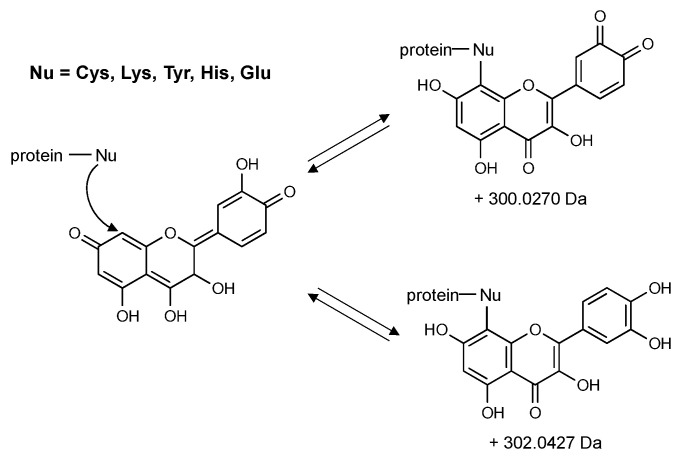
Proposed reaction scheme of nucleophilic protein residues with electrophilic flavonoid quinones. Expected characteristic mass shifts are shown for the quercetin quinone (**top**) and reduced quercetin (**bottom**).

Investigations of the protein adductome are still in a relatively early stage, and yet they provide a foundation of methods (mostly using LC-MS/MS) that could be used for the identification and characterization of flavonoid–protein adducts [71,72,117,118,119]. LC-MS/MS methods have already been used to identify protein–quinone adducts by examination and interpretation of fragmentation patterns in the spectra [71,72,120]. Protein–flavonoid adducts could be identified by analyzing tryptic digests of flavonoid–protein mixtures and then using established proteomics’ methodologies to identify peptides with mass shifts characteristic for a specific flavonoid. Strongly nucleophilic residues such as cysteine and lysine are probably the most reasonable locations to initially search for these types of adducts in proteins, but other residues such as tyrosine, histidine, and even glutamate may also act as adduct-forming sites. These adducts could be detected by searching a data set from LC-MS/MS analysis of a tryptic digest of quercetin treated protein. Quercetin adducts would have two probable characteristic mass shifts, either a reduced quercetin adduct with a monoisotopic mass of 302.0427 Da or a quercetin quinone adduct with a monoisotopic mass of 300.0270 Da (Figure 7). EGCG would be expected to yield adducts with monoisotopic masses of 458.0849 Da for the reduced species and 456.0693 Da for the oxidized quinone. Detailed analytical characterization of neutral loss species and diagnostic ions could be used to make identification of these types of adducts definitive.

An interesting example of the identification of protein-electrophile adducts is from the work of Richard van Breemen and colleagues who demonstrated that phenolic constituents from various plant sources form covalent adducts with cysteine residues in Keap 1 [111,121,122]. Although the electrophiles identified in their studies were chalcones and similar compounds rather than flavonoids, their work provides validation of the general approach of using tandem mass spectrometry to identify protein–electrophile adducts. They reported that, although Keap1 has 27 cysteine residues that may be able to form adducts with electrophiles, these residues do not react equally and Cys151 is preferentially alkylated over the other cysteine residues [121]. They also demonstrated the use of β-mercaptoethanol as a trapping reagent to identify reversible protein–electrophile adducts in cases where the electrophile dissociates from the protein too quickly for direct detection by mass spectrometry [122]. These results fit well with reports that some quinone electrophiles exhibit selectivity in forming adducts with specific sites on proteins rather than reacting equivalently with all nucleophilic residues [72,120] and that quercetin-glutathione adducts are reversible in aqueous solutions [91]. In all of these examples, the interactions between the electrophile and protein were covalent, further supporting the hypothesis that protein-flavonoid adducts may induce reversible biochemical changes even with the formation of covalent bonds.

## 5. Reactions and Mechanisms of Protein Oxidation

Like the other primary classes of biomolecules found in living cells (lipids, carbohydrates and nucleic acids), proteins can be oxidized, creating chemical changes that often directly impact their functions [68,69,123,124]. Protein oxidation was extensively studied in the 1990s in relation to aging and many high quality and highly cited review articles are available that describe the chemistry of amino acid oxidation in proteins as well as many of the biological consequences of these oxidations [13,125,126,127]. Because of this history, the fundamental concepts surrounding protein oxidation are relatively well understood. It is known that, while many different amino acids are susceptible to oxidation in proteins, cysteine, methionine, and tyrosine are much more frequently oxidized than other amino acid residues [68,128]. These residues can be oxidized by a variety of ROS and reactive nitrogen species (RNS), and, since it is well established that flavonoids can be disproportionate in aqueous solutions and form various ROS, it is plausible that flavonoids could promote the oxidation of specific residues in proteins and thus modify their functional properties as well. The metal-chelating properties of flavonoids could also contribute to the oxidation of protein residues either indirectly by generating ROS or through direct electron transfer processes at specific binding sites on a protein.

Cysteines are the most readily oxidized amino acids in proteins, a property that is often utilized as a regulatory feature of many proteins and cell signaling pathways. Cysteine disulfides are perhaps the most frequently cited example of cysteine oxidation products, but cysteine sulfinic acid (Cys-SOOH) is also a frequent oxidation product of cysteine and is a reversible post-translational modification that is known to regulate a variety of cell signaling processes [129,130]. As described above, it is known that specific signaling pathways such as the EpRE-mediated response can be activated through specific cysteine residues on Keap1 [103,104]. Although the formation of protein-flavonoid adducts is one possible mechanism of flavonoid activation of this pathway, it is also possible that flavonoids could directly oxidize or reduce these residues to induce the structural changes that lead to activation of the of signal pathway.

Methionine residues are also often oxidized to methionine sulfoxide [131]. Oxidation of methionine is a common enough occurrence that cells possess methionine sulfoxide reductase (MSR) enzymes to reverse this oxidative transformation [69,131]. MSRs provide important protection for cells against changes to protein structures that occur when methionine is oxidized and enable cells to use methionine oxidation as a switch to regulate specific signaling processes [132]. At the biochemical level, it is easy to imagine how conversion of a non-polar methionine residue to an amino acid with a polar, hydrogen-bond acceptor functional group could have dramatic consequences on the folding and function of a protein. There is also evidence demonstrating that reagents can oxidize specific methionine residues based on their steric interactions with a protein [128], which again suggests a possible mechanism for flavonoids to change protein structure and function by promoting oxidative changes to specific amino acid residues. If a flavonoid were to oxidize a methionine residue in a way that significantly changed protein activity, this change could be reversed through the activity of MSRs, thus creating a reversible mechanism of flavonoid bioactivity in cells.

Similar arguments could be made for the potential impact flavonoids could have on key tyrosine, lysine, and other residues in proteins. As described above, there are water molecules interacting with tyrosine and the flavonoid ligand in the inositol polyphosphate multikinase crystal structure [115] and in the Hck crystal structure [114]; the presence of water adjacent to tyrosine and lysine suggests that water could participate in the exchange of electrons and hydrogen between these residues and a flavonoid ligand. Considering the many examples described above of flavonoids binding to proteins in close proximity to these types of residues in the catalytic sites of different enzymes, it is again highly plausible that these compounds could exert inhibitory activity in enzymes by oxidizing specific residues. As with the identification of protein-flavonoid adducts, mass spectrometry is likely the most suitable method for searching for flavonoid-induced oxidative changes in proteins. Fortunately, there are many existing MS-based analytical methods for studying protein oxidation, and these could easily be adapted for studying protein-flavonoid interactions [133,134,135,136,137]. Tandem mass spectra analysis software such as SEQUEST [138] or MS Amanda [139] can be used to identify protein oxidation events in peptides. With appropriate use of isotopic labeling or using a label-free quantitation approach, the relative quantity of specific amino acids that might be oxidized through protein-flavonoid binding could be determined.

## 6. Summary and Conclusions

The vast number of papers related to flavonoid chemistry and bioactivity is a testament to the enduring interest these compounds hold in the imagination of the scientific community. However, it is this same enduring interest that creates a huge amount of noise in the literature and makes it difficult to find the much smaller number of papers that are advancing our understanding of flavonoid bioactivity. Despite this challenge, it is clear that progress is slowly being made. Careful design of studies and experiments to account for redox activity of flavonoids and their reactive quinone intermediates is revealing that these compounds can induce changes at specific sites on proteins that can explain the observed bioactivity. The following suggestions may provide useful guidance to improve mechanistic studies of flavonoid bioactivity:

1. Rigorously account for the various redox properties and reactive intermediates of flavonoids in experiments using analytical methods such as NMR or chemical additives such as glutathione and ascorbic acid;
2. Test precise hypotheses about protein-flavonoid interactions at specific amino acid residues;
3. Look for alternative interpretations of experimental results that account for protein-flavonoid interactions and go beyond the traditional paradigm of simple, non-covalent binding.
4. Avoid hypothesis or experiments that invoke the radical scavenging activity of flavonoids as a mechanistic explanation for observed bioactivity.

These suggestions are not the only approach to improving flavonoid bioactivity studies, but the widespread adoption of these or similar practices would go a long way toward facilitating new discoveries in the field.

I am confident that many exciting discoveries will be made related to mechanisms of flavonoid bioactivity in the near future. Whether it is the identification of protein-flavonoid adducts, flavonoid-induced protein oxidation, the bioactivity of previously unstudied products of flavonoid metabolism by gut microbes, or other mechanisms that have yet to be discovered, opportunities for progress abound. Flavonoid bioactivity studies will continue to be a challenging field to work in due to the complexities of flavonoid chemistry in aqueous systems, but the rewards will be well worth the effort. Beyond the knowledge gained of how flavonoids directly impact human health, there are additional benefits to be gained from identifying specific mechanisms of flavonoid–protein interactions. Novel systems for modifying protein activity may be gained by identifying previously known regulatory mechanisms that depend on specific amino acid residues. In light of the poor bioavailability of flavonoids and the fact that as much as 70% of dietary flavonoids may be metabolically degraded by gut microbes prior to absorption by intestinal endothelial cells, it seems that flavonoids have very little potential as direct therapeutic agents [16,18,77], but flavonoids can continue to act as useful probes of new biochemistry. It seems very safe to predict that many new and exciting discoveries are just around the corner.
